# Deciphering the Link Between Gut Bacterial Community Structure of Forest Musk Deer (*Moschus berezovskii*) and Musk Traits

**DOI:** 10.3390/vetsci13090957

**Published:** 2026-09-13

**Authors:** Jun Guo, Hang Jie, Diyan Li, Tao Wang, Si Deng, Caiyi Peng, Chengli Zheng, Xiuxiang Meng, Chenglu Zhang, Zhongxian Xu

**Affiliations:** 1 Key Laboratory of Southwest China Wildlife Resources Conservation (Ministry of Education), China West Normal University, Nanchong 637009, China; is_guojun@163.com (J.G.); dengdum@163.com (S.D.); 17390257036@163.com (C.P.); 2 Chongqing Institute of Medicinal Plant Cultivation, Chongqing University of Chinese Medicine, Chongqing 408435, China; jiehangisgood@126.com; 3 School of Pharmacy, Chengdu University, Chengdu 610106, China; lidiyan@cdu.edu.cn; 4 College of Basic Medicine, Chengdu University, Chengdu 610106, China; wanttao@hotmail.com; 5 Sichuan Institute of Musk Deer Breeding, Sichuan Institute for Drug Control, Chengdu 611830, China; zhengcl@scidc.org.cn; 6 School of Resources and Environment, Aba Teachers University, Wenchuan 623002, China; meng2014@ruc.edu.cn

**Keywords:** forest musk deer (*Moschus berezovskii*), gut bacterial microbiota, musk production, musk quality, 16S rRNA

## Abstract

Musk, a precious traditional Chinese medicine, is secreted by the male forest musk deer (*Moschus berezovskii*). The gut microbiota plays an essential role in host metabolism, yet its relationship with musk traits remains poorly understood. Here, 16S rRNA sequencing of 89 fecal samples was integrated with musk quality (color and moisture) and yield data to investigate how fecal bacterial communities influence these traits. Distinct microbial consortia were associated with musk traits. Aromatic and sulfur-metabolizing taxa (*norank_f_Actinomycetaceae*, *Nocardioides*, *Rhodococcus*, and *Mailhella*) were associated with degradation of aromatic compounds and sulfur-containing amino acids, potentially contributing to muscone and other bioactive aroma components. Short-chain fatty acid (SCFA) producing taxa (*Blautia*, *Phascolarctobacterium*, *Oscillospiraceae UCG-005*, *unclassified_f_Lachnospiraceae*, *Anaerovorax*, *Parabacteroides*, *Prevotellaceae_UCG-003*, *Christensenellaceae R-7 group*, and *Bacteroides*) were capable of fiber decomposition and may supply energy for steroid synthesis via SCFAs, favoring high-quality musk formation. *Ketogulonicigenium vulgare* was linked to anti-inflammatory polypeptides and steroids. These potential indicators may facilitate early selection of high-yielding deer, inform probiotic/prebiotic interventions, and support dietary strategies to improve gut health and musk productivity, thereby reducing poaching and promoting the sustainable conservation of this endangered species.

## Simple Summary

The forest musk deer (FMD) is a small ruminant that produces musk, a precious substance used in traditional medicine for thousands of years. However, these deer are endangered, and there is a growing gap between the demand for musk and its supply. In this study, we explored whether the bacteria living in the deer’s gut could influence the quality and amount of musk they produce. By analyzing fecal samples from 89 male deer, results showed that certain groups of gut bacteria are closely linked to better musk traits. Some bacteria help produce the fragrant compounds that give musk its unique smell, while others help break down plant fiber to provide energy for musk formation. These findings may allow farmers to identify high-producing deer at an early stage and to improve musk yield through better diets or probiotics. Enhancing farm-based musk production could reduce poaching, protect wild populations, and help meet the demand for this precious medicine in a sustainable way.

## 1. Introduction

The gut microbiota of livestock break down and ferment the ingested feed into nutrients, which are utilized to produce meat, milk, and eggs [1,2]. A well-balanced microbiome is a key regulator of animal physiology, improving nutrient absorption and metabolic efficiency [3,4]; and is also involved in regulating immunity, promoting intestinal development, and defense against pathogens [5]. Disruption of the “host-gut microbiota” system can impair the host’s physiological functions and even induce various diseases [6]. In *Moschus berezovskii* (forest musk deer: FMD), probiotics (e.g., *Lactobacillus casei* Zhang, *Lactobacillus plantarum* P-8) interact with the host by acting as a protective barrier against pathogens such as *Escherichia coli*, *Citrobacter freundii*, and supporting the growth of beneficial taxa (e.g., *Bifidobacterium*) [7]. A higher Firmicutes/Bacteroidetes ratio is associated with improved gut health and reduced incidence of diarrhea; especially, *Akkermansia* and *Lachnospira* enriched under a high-energy diet significantly enhanced growth performance [8]. Additionally, the gut microbiota has been recognized as a critical modulator of androgen synthesis and metabolism [9], suggesting potential crosstalk with reproductive hormone-dependent traits like musk secretion. Androgens, specifically testosterone, bridge the endocrine system and musk formation; they are hypothesized to positively correlate with musk production and fluctuate with microbial composition and muscone content during musk secretion [10]. Firmicutes, Bacteroidetes, and Proteobacteria were microbial taxa shared between the gut and musk pouch [10,11,12,13]; individuals with elevated abundance of Firmicutes were able to maximize energy acquisition from fiber and polysaccharide, which might affect their ability to secrete musk [14]. In particular, *Fibrobacteres*, Tenericutes, and *Verrucomicrobia* were significantly enriched in high-yielding FMD, suggesting the gut microbiota may regulate musk secretion through their Short-chain fatty acids (SCFAs) metabolites, which indirectly influence musk composition via interplay with steroid metabolism [15]. Meanwhile, levels of steroids (estradiol, testosterone, cortisol, and corticosterone) in the high-yielding group were significantly higher than those of the low-yielding group, illustrating the regulation of musk production by the gut microbiota through androgen/steroid metabolism [16].

Musk, the dried secretion from the musk sac located between the navel and genitals of mature male FMD [17,18], is a high-value resource with irreplaceable applications in traditional Chinese medicine (for resuscitation, blood circulation promotion, anti-inflammation, and pain relief) and the perfume industry as a premium fixative [19,20]. However, over decades of large-scale hunting and habitat loss, the FMD has become an endangered species, leading to a severe scarcity of natural musk [21]. Although China has expanded captive breeding programs for FMD from the 1990s to the 2020s, natural musk faces a persistent demand-supply imbalance in global markets [22]. This creates an urgent gap between supply and demand [21,23], which is influenced by factors such as hormone levels [12,24,25], mating history [26], and microbial fermentation [10,26]. Although progress has been made in understanding the physiological process of musk secretion, the specific role of microbiota in mediating musk production remains unclear. This knowledge gap limits the development of targeted strategies to alleviate the persistent musk scarcity.

The quality of mature musk directly affects the animal’s ability to communicate chemically and attract mates [26,27]. Higher-quality musk commands greater economic value, further underscoring the importance of understanding its biological formation [15]. Initially, the glandular epithelial cells secrete immature, milky white liquid with a fishy odor, this liquid is then transported via ducts to the musk sac, where it gradually transforms into a mature, brown solid with a characteristic aroma [27,28,29]. Traditionally, high-quality musk is characterized by a dry, solid appearance with a red or dark-brown color. Visual inspection of color and appearance is insufficient to accurately assess musk quality, as musk undergoes periodic changes throughout secretion, including seasonal variations in color, morphology, water content, and chemical composition [27]. These changes likely reflect shifts in physiological status and microbiota variation [26,27]. Based on muscone content (a critical bioactive component), musk is further classified into normal (brown solid with fragrant aroma) and abnormal (white semisolid or black muddy with pungent odor) types [27]. However, the mechanisms underlying these quality differences remain unelucidated, underscoring the need for systematic classification to advance research on musk traits and their relevance to animal health and productivity.

Given that captive individuals exhibit altered microbial composition and remain susceptible to gut diseases compared to wild populations, establishing the link between gut microbiota and musk secretion is directly relevant to species conservation [30,31]. During musk secretion, processes such as stabilization of organic and inorganic components, reduction in water content, darkening, and solidification occur; the composition of the intestinal flora shapes the final phenotype of mature musk [16]. It was reported that fungal genera associated with disease, such as *Colletotrichum* and *Apiotrichum*, are prevalent in musk deer producing abnormal musk, and gut fungal dysbiosis correlates with reduced musk yield and quality [13]. 16S rRNA sequencing was conducted on 89 fecal samples of male musk deer to further explore the relationship between the composition of fecal bacteria and musk traits, and to systematically evaluate their musk production (yield) and quality (color, morphology). Understanding these relationships can inform non-invasive microbiota-based strategies to improve captive health and musk production, thereby reducing reliance on wild populations and supporting the long-term sustainability of captive breeding as a conservation tool.

## 2. Materials and Methods

### 2.1. Animal Sampling and Sample Collection

A total of 89 fecal samples were collected from 89 healthy captive male FMD aged 4–12 years at the Sichuan Institute of Musk Deer Breeding (Sichuan, China). All animals were raised under captive management conditions, including individual housing, dietary standardization and quality control, regular pathogen screening (for *Escherichia coli*, *Clostridium perfringens*, *Brucella*, and *Clostridium tetani*), and maintenance of appropriate temperature and humidity, as described in a previous study [13]. Animals that tested positive for any pathogen were excluded from sampling to prevent confounding effects on the gastrointestinal microbiota. Both fecal and musk samples were collected at the end of October 2024, which corresponds to the musk maturation period. For each individual deer, feces and musk were collected on the same day to ensure a close temporal association between gut microbiota composition and musk traits. To avoid environmental contamination and to guarantee that each fecal sample originated from a single deer, feces were collected directly from the indoor housing pens (approximately 35 m^2^ per animal) rather than from the shared outdoor exercise areas. Each indoor pen was cleaned daily before the morning collection. Freshly defecated droppings were gathered at 6:00–6:30 a.m. to minimize exposure to ambient bacteria and to preserve microbial integrity. Musk was collected non-invasively from the same individuals using a sterile long-handled spoon inserted into the musk pod after disinfection of the surrounding skin, which does no harm to the deer or affect subsequent musk secretion. Following collection, the samples were immediately frozen on dry ice during transport, and subsequently stored at −80 °C until further processing. A total of 89 male forest musk deer were included in this study, of which 83 produced musk, and 6 did not. All experiments were conducted in accordance with guidelines approved by China West Normal University (Approval ID: 2024LLSC0070, Approval Date: 1 July 2024), and every effort was made to minimize the suffering of the animals.

Musk samples were visually inspected under standardized lighting by three independent trained evaluators. Colors were classified into six categories: white, yellow-brown, red-brown, dark-brown, brown, and black, following the established system [13]. Morphology was categorized into four groups (powder, strip, paste, and mud-like) based on physical consistency. To validate this classification, moisture content was measured gravimetrically: approximately 1 g of musk was dried at 105 °C for 4 h to constant weight, and moisture percentage was calculated as (wet weight − dry weight)/wet weight × 100%. Musk yield was recorded as the average wet weight of three consecutive years of musk collected from each individual. Musk quality was defined based on color and moisture content, following established criteria that darker-colored (red-brown to dark-brown) and lower-moisture (powder or strip) musk contains higher concentrations of bioactive compounds such as muscone and is therefore considered higher quality [13,32].

### 2.2. Bacterial DNA Isolation and 16S rRNA Sequencing

Total microbial genomic deoxyribonucleic acid (DNA) was extracted from the fecal samples using the E.Z.N.A.^®^ Soil DNA Kit (Omega Bio-tek, Norcross, GA, USA) according to the manufacturer’s instructions. DNA quality was verified through 1% agarose gel electrophoresis, and concentration and purity were determined via a NanoDrop2000 (Thermo Scientific, Waltham, MA, USA). The V3–V4 hypervariable region of the bacterial 16S ribosomal RNA (16S rRNA) gene was amplified via a T100 Thermal Cycler PCR (polymerase chain reaction) thermocycler (Bio-Rad, Hercules, CA, USA) using the universal primers 338F (ACTCCTACGGGAGGCAGCAG) and 806R (GGACTACHVGGGTWTCTAAT). The 16S rRNA gene was amplified by PCR (95 °C for 3 min, followed by 27 cycles of 95 °C for 30 s, 55 °C for 30 s, and 72 °C for 45 s, and a single extension of 8 min at 72 °C, ending at 4 °C). The PCR product library was generated via the NEXTFLEX Rapid DNA-Seq Kit and sequenced on the Illumina NextSeq2000 platform (Illumina, San Diego, CA, USA).

### 2.3. Raw Data Denoising and Species Annotation

The obtained low-quality raw reads were processed using fastp (version 0.19.6) s for quality control [33] and assembled with FLASH (version 1.2.11) [34]. Subsequently, sequence optimization and denoising were performed using the Deblur [35] plugin in QIIME 2 (version 2.2.0) [36], generating amplicon sequence variants (ASVs). On the basis of ASV information, we conducted the analysis using the vegan (version 2.4.3) package in R (version 3.3.1). For taxonomic classification, the ASVs were analyzed against the Silva 16S rRNA database (version 138) using QIIME 2’s Blast classifier. To obtain the taxonomic information corresponding to each ASV, the classify-sklearn algorithm (naïve Bayes classifier) was employed for taxonomic annotation of ASV representative sequences at multiple classification levels. Finally, functional prediction of 16S rRNA genes was performed using PICRUSt2 [37].

### 2.4. Alpha and Beta Diversity

To determine whether gut microbial richness and diversity differ across musk quality categories, the α diversity analysis was conducted to evaluate species diversity complexity through two indices (ACE: abundance-based coverage estimator and Shannon), implemented in Mothur with the default values (version 1.30.2) [38]. The ACE index reflects community richness, whereas the Shannon index reflects community diversity. Statistical comparisons of α diversity indices between musk color and moisture content groups were conducted using the Kruskal-Wallis rank sum test in R software (version 3.3.1). This approach is robust against non-normal distributions and heteroscedasticity, and it is widely recommended for multi-group comparisons of alpha diversity in microbiome studies.

The β-diversity analysis was utilized to further assess the differences among samples. The similarity among the microbial taxa in different samples was determined by principal coordinate analysis (PCoA) based on unweighted UniFrac distances and Bray-Curtis distances via the Vegan v2.5-3 package. PERMANOVA (permutational multivariate analysis of variance) via the adonis2 (9999 permutations) function in R (version 3.3.1) was used to test for overall differences in community composition, with musk yield included as a covariate to assess the independent effects of color and morphology grouping.

### 2.5. Microbial Characterization Index

Although all sampled animals were clinically healthy based on regular pathogen screening and absence of disease symptoms, the Gut Microbiome Health Index (GMHI) [39] was applied as an exploratory tool, not as a validated measure of gut health or disease propensity in musk deer, to assess potential subclinical differences in gut microbial ecology associated with musk production capacity. GMHI is a robust index that evaluates the likelihood of a disease-prone microbiome based on species-level taxonomic features. To evaluate whether GMHI values are associated with musk quality, the FMD that did not produce musk were categorized as non-musk-secreting group (*n* = 6), and the remaining 83 musk deer as the musk-secreting group, following the classification described in previous study [13], GMHI values were calculated for each sample using Python (version 2.7), and differences between the two groups were compared using the Wilcoxon rank-sum test, a non-parametric method suitable for comparing groups with unequal sample sizes. This approach allowed us to identify potential differences in species-level microbial profiles associated with musk production capacity. Because the non-musk-secreting group comprised only six individuals, this limits the statistical power of this comparison and emphasizes the need for validation in larger cohorts. This should be interpreted with caution and considered exploratory rather than diagnostic.

### 2.6. Bacterial Difference Analysis and Function Prediction

Genus-level taxonomic comparisons between the musk color and morphology groups were analyzed using the Kruskal-Wallis test followed by Dunn’s post hoc test with Benjamini-Hochberg correction for multiple comparisons in R (version 3.3.1, stats package). ASV sets were then constructed separately for the differential bacteria in the color groups and the morphology groups. The Kyoto Encyclopedia of Genes and Genomes (KEGG) metabolic pathways and enzymes of the differential bacteria in the color groups and morphology groups were predicted on the basis of ASV representative sequences using PICRUSt2 (Phylogenetic Investigation of Communities by Reconstruction of Unobserved States) [37]. Multiple comparisons of the predicted functional profiles were conducted within each group using the Kruskal-Wallis test.

### 2.7. Analysis of Correlations Between Gut Bacteria and Musk Production

Pearson correlation analyses were performed to evaluate associations between the relative abundance of candidate gut bacterial genera and musk yield using the cor.test function (two-tailed) in R (v3.3.1). In total, 10 genus-trait pairs were tested. Benjamini-Hochberg false-discovery-rate (BH-FDR) correction was applied to account for multiple testing. Correlations with BH-FDR-adjusted *p* < 0.05 were considered statistically significant. The heatmaps were generated using the pheatmap package in R (version 3.3.1) to visualize the relationships between gut bacteria and musk yield. Additionally, on the basis of the average abundance of the gut microbiota in the musk color and morphology groups of the FMD, the expression patterns of the gut microbiota were analyzed using the Mfuzz R package (version 2.66.0) [40].

### 2.8. Declaration of AI Tools Usage

During the preparation of this manuscript, the literature search was performed using the DeepSeek large language model as an auxiliary tool to query multiple bibliographic databases (e.g., PubMed, Web of Science, Google) via application programming interfaces (APIs) and to aggregate candidate references based on predefined keywords; Every cited reference was independently verified against the original sources. Grammatical refinement was assisted by DeepSeek as well; all AI-generated suggestions were critically reviewed and edited by the authors, who take full responsibility for the final content. No AI tools were used for data analysis, result generation, or scientific interpretation.

## 3. Results

### 3.1. Musk Quality Traits and 16S rRNA Sequencing Data of the Gut Bacteria

**Figure 1:**
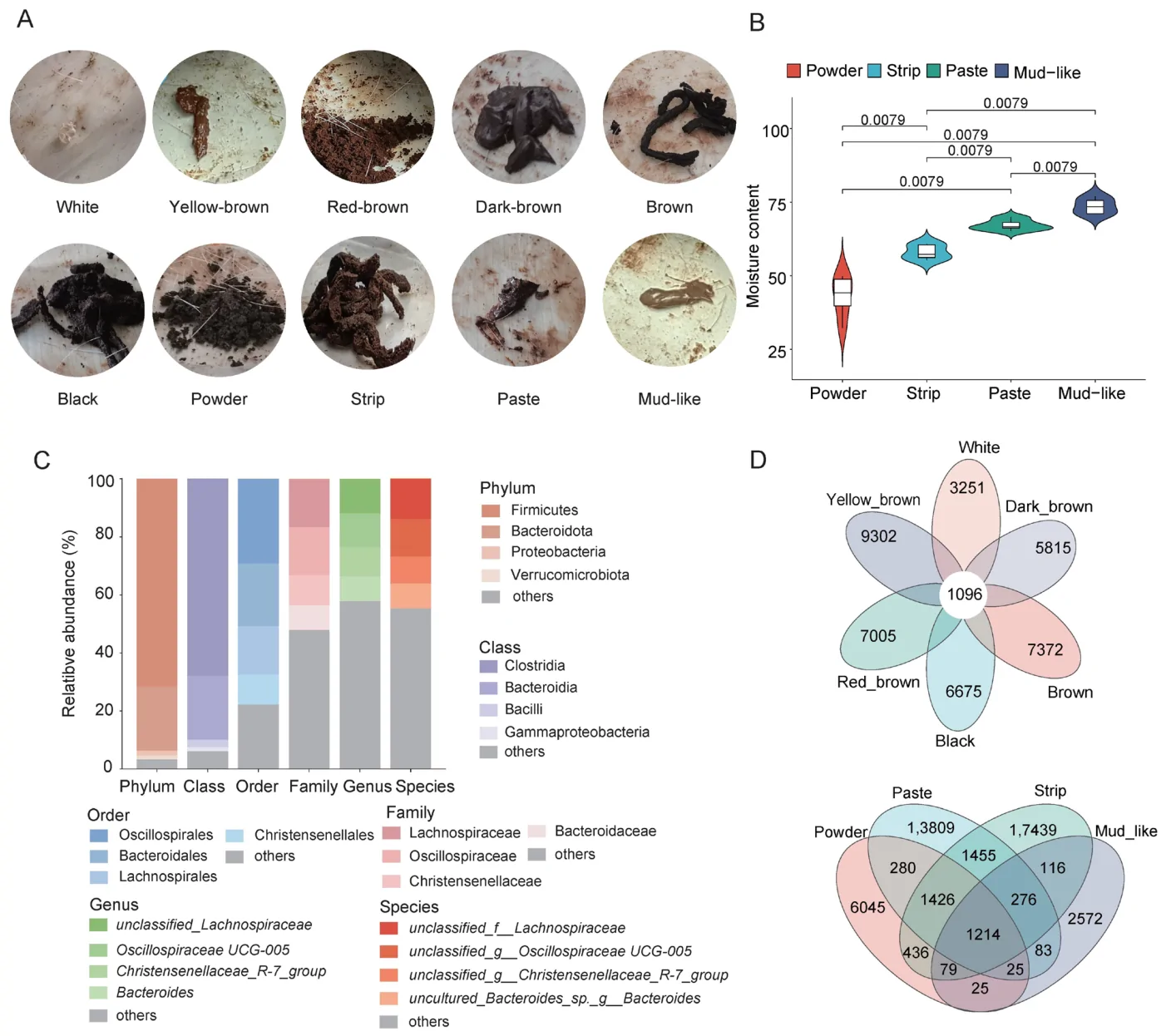
Musk quality trait characterization and gut bacterial profiling in FMD. (**A**) Representative images of musk exhibiting distinct color and morphological phenotypes. (**B**) Differences in moisture content across musk morphology groups. (**C**) Taxonomic distribution of dominant gut bacteria in FMD. (**D**) Venn diagram of shared ASVs between musk color groups (**upper**) and morphological groups (**lower**).

Of the 89 deer, 83 that produced musk were included in subsequent analyses of musk quality traits (color, morphology, and yield); the six non-secreting individuals were included only in the GMHI comparison (Supplementary Table S1). Musk quality is characterized by diverse color and morphological traits, which vary with individual differences and seasonal dynamics. As shown in Figure 1A i.e., musk color: white *n* = 7, yellow-brown *n* = 18, red-brown *n* = 15, dark-brown *n* = 12, brown *n* = 17, black *n* = 14). The musk morphology was grouped based on their moisture content: powder (lowest moisture, *n* = 13), strip (*n* = 30, including 7 white musk), paste (*n* = 35), and mud-like (highest moisture, *n* = 5). Statistical analysis confirmed significant differences in moisture content across morphological groups (*p* = 0.0079), with powder musk exhibiting the lowest moisture and mud-like musk the highest (Figure 1B).

To characterize gut bacterial communities associated with musk traits, 16S rRNA sequencing was performed on 89 fecal samples from FMD. After quality control, 5,385,831 optimized sequences were generated (mean = 60,515 reads per sample. Denoising yielded 48,024 amplicon sequence variants (ASVs; mean = 1109 ASVs per sample) and 3,101,064 valid reads (mean = 34,843 reads per sample; Supplementary Tables S2 and S3). Taxonomic annotation revealed 1 kingdom, 23 phyla, 40 classes, 98 orders, 184 families, 457 genera, 863 species, and 48,024 ASVs. At the phylum level, the gut microbiota was dominated by Firmicutes (71.52%), Bacteroidota (22.26%), Proteobacteria (1.73%), and Verrucomicrobiota (1.20%). At the genus level, core taxa included *unclassified_Lachnospiraceae* (11.91%), *Oscillospiraceae UCG-005* (11.54%), *Christensenellaceae_R-7_group* (10.1%), and *Bacteroides* (8.75%). Species-level analysis highlighted *unclassified_f_Lachnospiraceae* (11.89%), *unclassified_g_Oscillospiraceae UCG-005* (11.01%), *unclassified_g_Christensenellaceae R-7 group* (6.27%), and *uncultured_Bacteroides_sp._g_Bacteroides* (5.88%) as dominant taxa (Figure 1C, Supplementary Table S3). ASV-level analysis further revealed shared microbial signatures: 2.78% (1096/39,420) of ASVs were common across color groups, and 3.05% (1214/39,865) were shared across morphological groups (Figure 1D), suggesting conserved gut bacterial taxa associated with musk trait variation.

### 3.2. Composition of the Gut Bacteria Across the Musk Color and Morphology Groups

**Figure 2:**
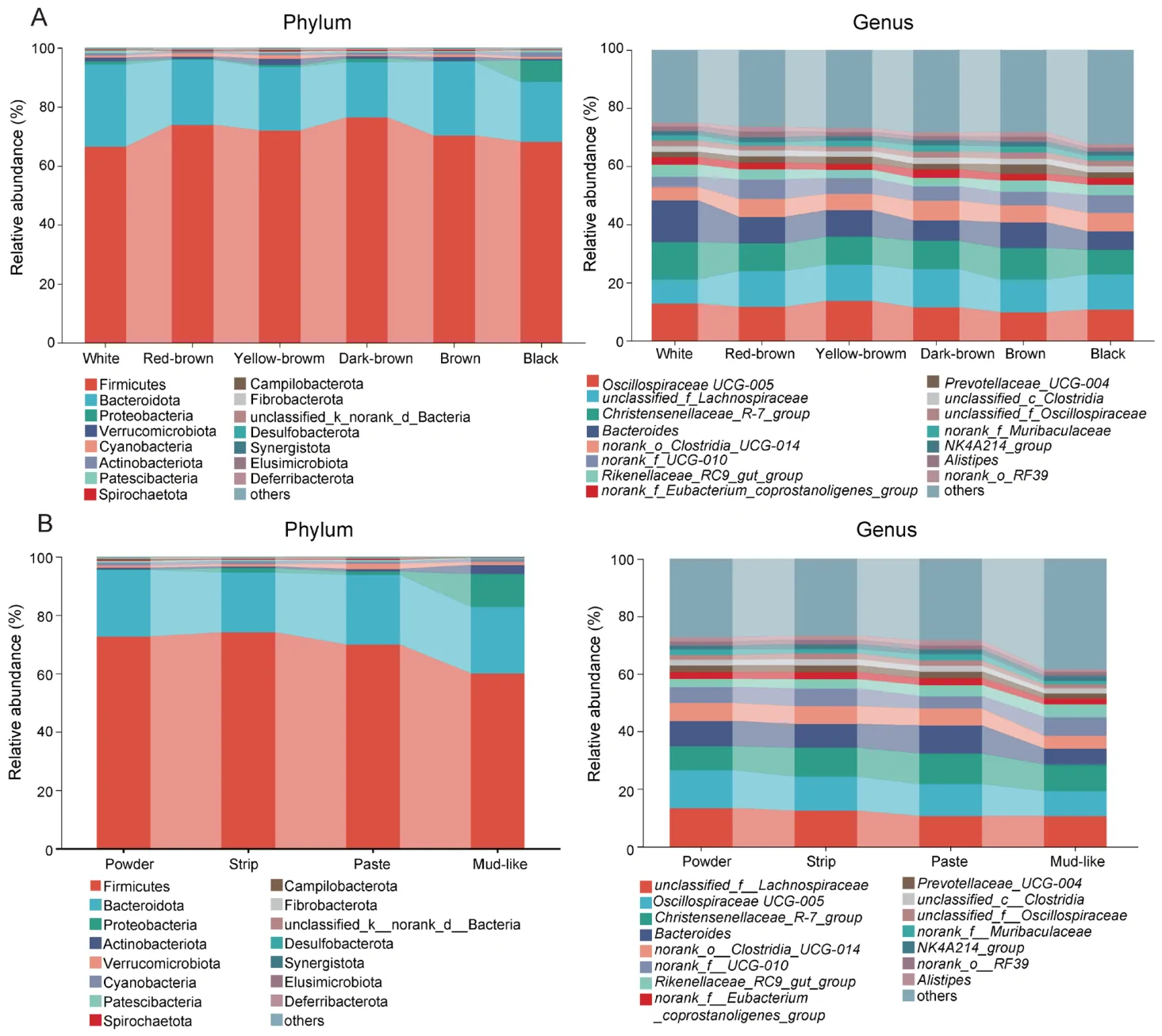
Gut bacterial taxonomic shifts across musk quality trait groups. (**A**) Stacked bar plots of dominant gut bacteria at the phylum (**left**) and genus (**right**) level in the musk color group. (**B**) Stacked bar plots of dominant gut bacteria at the phylum (**left**) and genus (**right**) level in the musk morphology group.

The bacterial community composition across musk color and morphology groups was analyzed to characterize gut microbiota variation associated with musk quality traits. Firmicutes and Bacteroidota accounted for more than 90% of the microbiota at the phylum level (Figure 2). Firmicutes and Bacteroidota together accounted for the smallest proportion in black musk (68.20% and 20.23%, respectively) and the highest in mud-like musk (60.02% and 22.81%). The composition of Bacteroidota was significantly different in brown vs. dark-brown (*p* = 0.048), and Firmicutes was significantly different in paste vs. mud-like (*p* = 0.034). The Firmicutes/Bacteroidetes (F/B) ratio ranged from 2.38 in white to 4.06 in the dark-brown group, and varied from 2.63 in the mud-like to 3.61 in the paste group (Supplementary Table S4). But only in the comparison of the strip vs. paste group was there a significant difference (*p* = 0.034) (Supplementary Table S5). At the genus level, the top 3 abundant taxa were Firmicutes: *Oscillospiraceae UCG-005* (11.67%), *unclassified_f_Lachnospiraceae* (11.61%), and *Christensenellaceae_R-7_group* (10.17%), followed by *Bacteroides* (9.11%) (Figure 2). Among them, *Oscillospiraceae UCG-005* was significant different in brown vs. red-brown (*p* = 0.007) comparison, significant differences of *unclassified_f__Lachnospiraceae* exhibited in darkbrown vs. white (*p* = 0.034) and yellow-brown vs. white (*p* = 0.042) comparisons. The relative abundance of *Bacteroides* increased from 6.31% in black to 14.39% in white; it was significantly different between black and brown/red-brown/yellow-brown groups, with *p* = 0.044, 0.017, and 0.044, respectively. In the morphology group, the lowest abundance was in the mud-like (8.55%, 10.66%, 9.31%) and the highest was in the powder (13.13%, 13.45%, 8.28%) group of the first three genera, respectively. *Bacteroides* ranged from 5.56% in mud-like to 14.39% in white (Supplementary Tables S4 and S5).

### 3.3. Gut Bacterial Diversity and Health Indices

**Figure 3:**
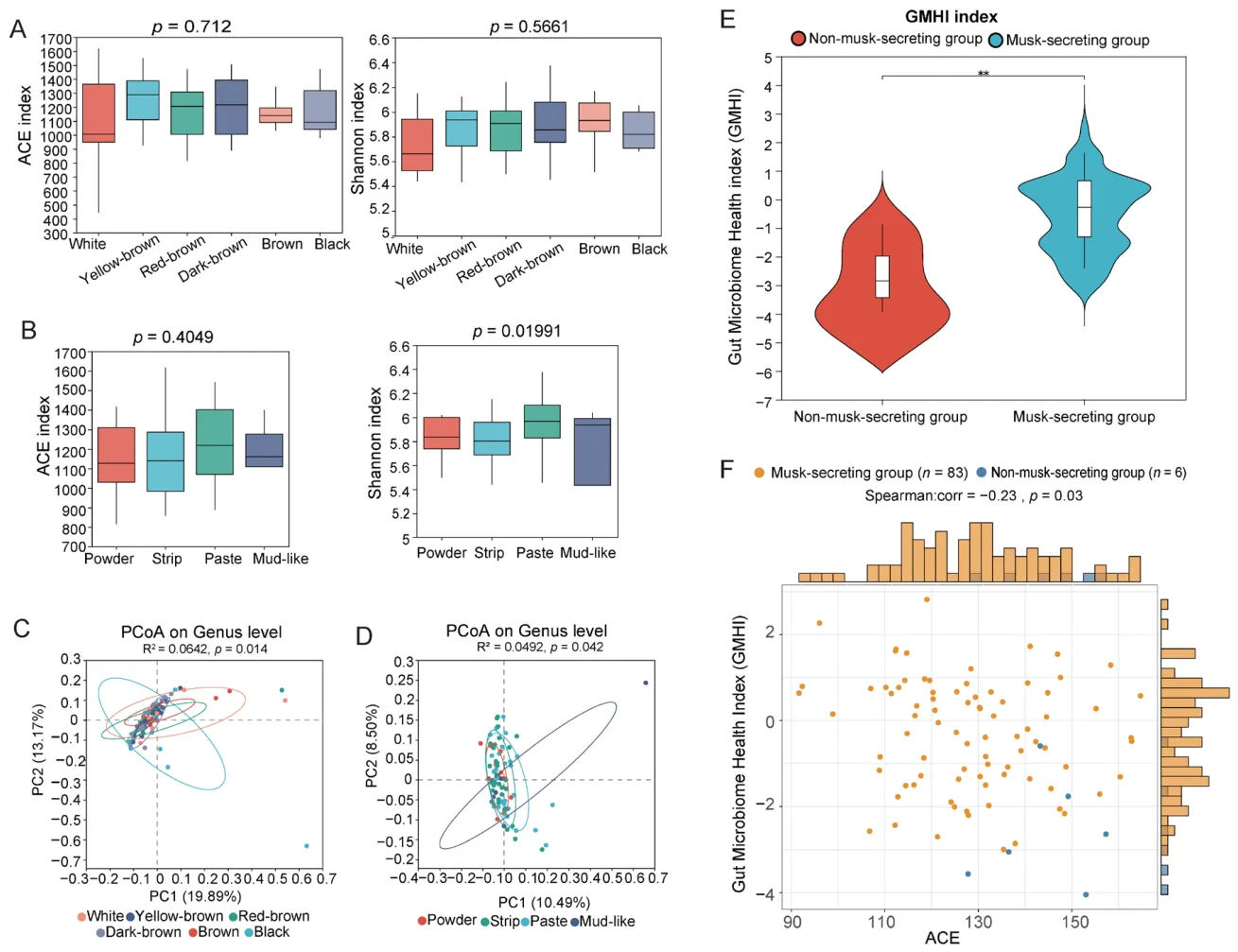
Gut microbiota diversity and health index across musk color and morphology groups in forest musk deer (FMD). (**A**) Abundance-based coverage estimator (ACE, richness) and Shannon (diversity) indices across musk color groups. (**B**) ACE (richness) and Shannon (diversity) indices across musk morphology groups. (**C**) Principal coordinates analysis (PCoA) plots based on genus-level community composition across musk color groups. (**D**) PCoA plots based on genus-level community composition across musk morphology groups. (**E**) Comparing gut microbiome health index (GMHI) values between non-musk-secreting and musk-secreting groups. (**F**) The correlation between ACE and GMHI in non-musk-secreting and musk-secreting groups. Pairwise comparisons were performed using the Wilcoxon rank-sum test; the significance levels are indicated as: 0, ** *p* < 0.01.

The α- and β-diversity of gut microbiota across musk color and morphology groups in FMD were analyzed to characterize biodiversity differences. For the α diversity, no significant differences were observed across musk color groups (ACE: *p* = 0.71; Shannon: *p* = 0.57) (Figure 3A). In morphology groups, ACE indices did not differ (*p* = 0.40), while Shannon indices showed marginal multigroup significance (*p* = 0.02) but no pairwise differences; strip-shaped musk exhibited the highest richness (Figure 3B). For β-diversity, principal coordinate analysis (PCoA) revealed clustering of color groups (*R*^2^ = 0.064, *p* = 0.014) and morphology groups (*R*^2^ = 0.049, *p* = 0.042), indicating intra-group compositional similarity (Figure 3C,D). PERMANOVA with yield included as a covariate showed that the independent effects of color grouping (Pr(>F) = 0.098) and morphology grouping (Pr(>F) = 0.066) on microbial community composition were not significant after accounting for yield. Suggesting that the non-significant PERMANOVA results were not confounded by unequal group dispersion.

The GMHI for non-musk-secreting (*n* = 6) and musk-secreting (*n* = 83) groups was calculated to explore links between gut health and musk secretion. GMHI values were significantly higher in the musk-secreting group than in the non-secreting group, indicating that non-secreting deer harbored a gut microbiome with species-level features more similar to those associated with poor health, despite lacking clinical symptoms. To further examine whether this difference was independent of microbial richness, the correlation analysis between GMHI and the ACE richness index was performed using all samples. A significant negative correlation was observed between GMHI and ACE (Spearman’s *r* = −0.23, *p* = 0.03), and GMHI better distinguished secretion status than ACE alone (Figure 3E,F). These findings suggest that gut microbial community composition, rather than richness alone, is more closely linked to musk production capacity.

### 3.4. Differential Taxa and Functional Profiling of Gut Bacteria Across Musk Trait Groups

**Figure 4:**
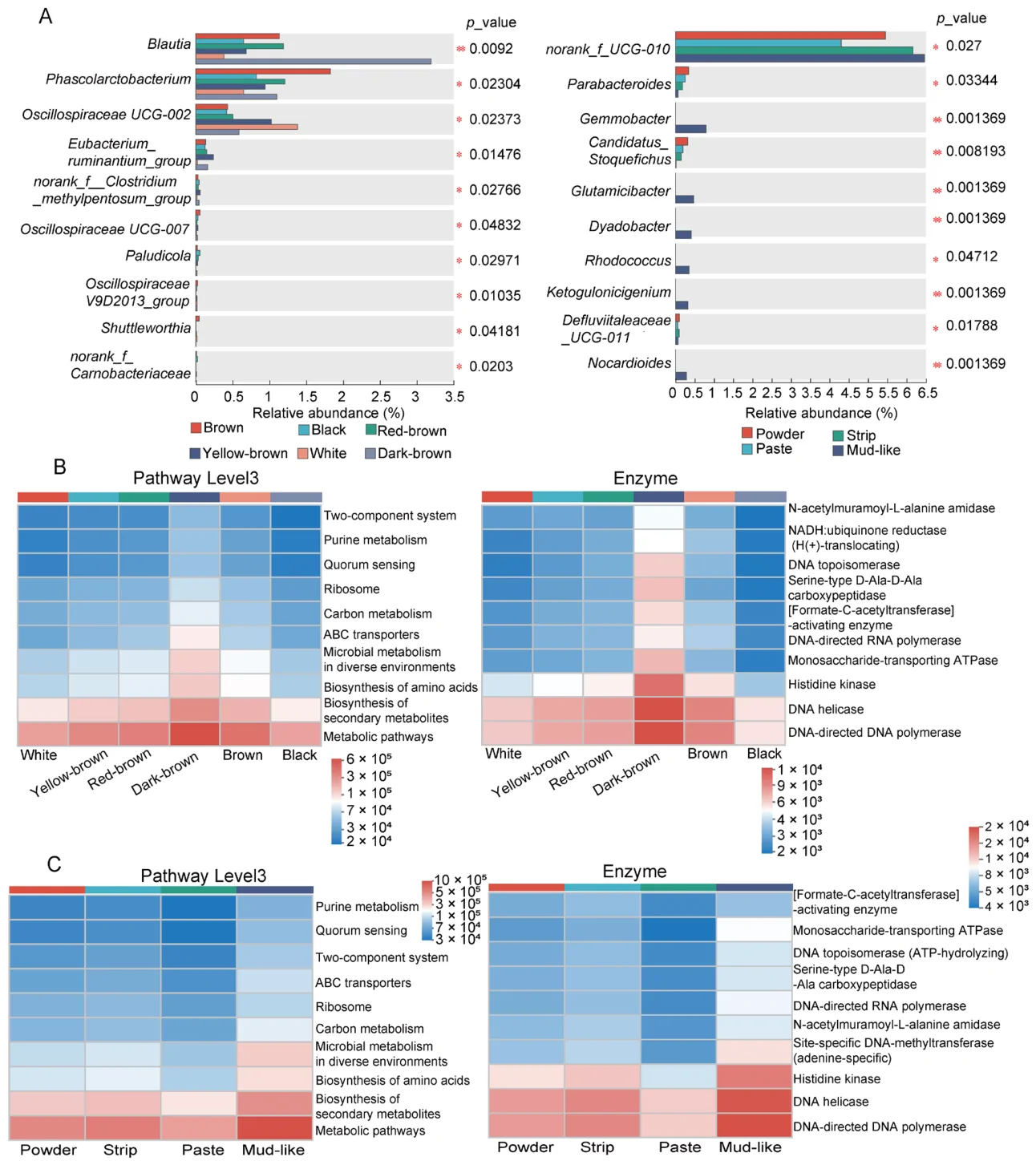
Differential gut bacterial taxa and functional signatures across musk traits. (**A**) Multigroup comparisons of gut bacteria in musk color (**left**) and morphology (**right**) groups. * *p* < 0.05, ** *p* < 0.01. (**B**) Predicted metabolic pathways (**left**) and enzymes (**right**) in color groups. (**C**) Predicted metabolic pathways (**left**) and enzymes (**right**) in morphology groups.

The multigroup differential abundance analyses (Kruskal-Wallis rank-sum test) on gut bacteria from musk color and morphology groups were performed to identify candidate bacterial taxa that may serve as potential phenotype indicators. Among the top 10 abundant taxa, color groups were distinguished by taxa such as *Blautia*, *Phascolarctobacterium*, *Oscillospiraceae UCG-002*, and the *Eubacterium ruminantium group*. Morphology groups were characterized by *norank_f_UCG-010*, *Parabacteroides*, *Gemmobacter*, and *Candidatus_Stoquefichus*. Notably, taxa including *Gemmobacter*, *Glutamicibacter*, *Dyadobacter*, *Rhodococcus*, *Ketogulonicigenium*, and *Nocardioides* were uniquely enriched in mud-like musk (Figure 4A), suggesting specialized roles in moisture-driven maturation.

We inferred metabolic functions and enzymatic activities from ASV profiles, using PICRUSt2. In color groups, dominant pathways included global metabolism (ko01100), secondary metabolite biosynthesis (ko01110), amino acid biosynthesis (ko01230), and microbial metabolism in diverse environments (ko01120). The GMHI communities of the dark-brown musk groups were additionally enriched carbon metabolism (ko01200) and ATP-binding cassette (ABC) transporters (ko02010), alongside enzymes like DNA-directed DNA polymerase (EC2.7.7.7), DNA helicase (EC3.6.4.12), histidine kinase (EC2.7.13.3), and monosaccharide-transporting adenosine triphosphatase (ATPase) (EC3.6.3.17), implying microbial involvement in pigmentation and structural maturation (Figure 4B). In morphology groups, core pathways mirrored color groups (global metabolism, secondary metabolite biosynthesis) but with amino acid biosynthesis and microbial metabolism pathways specifically enriched in mud-like musk. Key enzymes (histidine kinase, DNA helicase, DNA-directed DNA polymerase) were also abundant in mud-like musk, aligning with moisture-dependent functional specialization (Figure 4C). These findings highlight microbial metabolic signatures underlying musk quality variation.

### 3.5. Yield-Associated Bacteria and Quality-Driven Microbes

**Figure 5:**
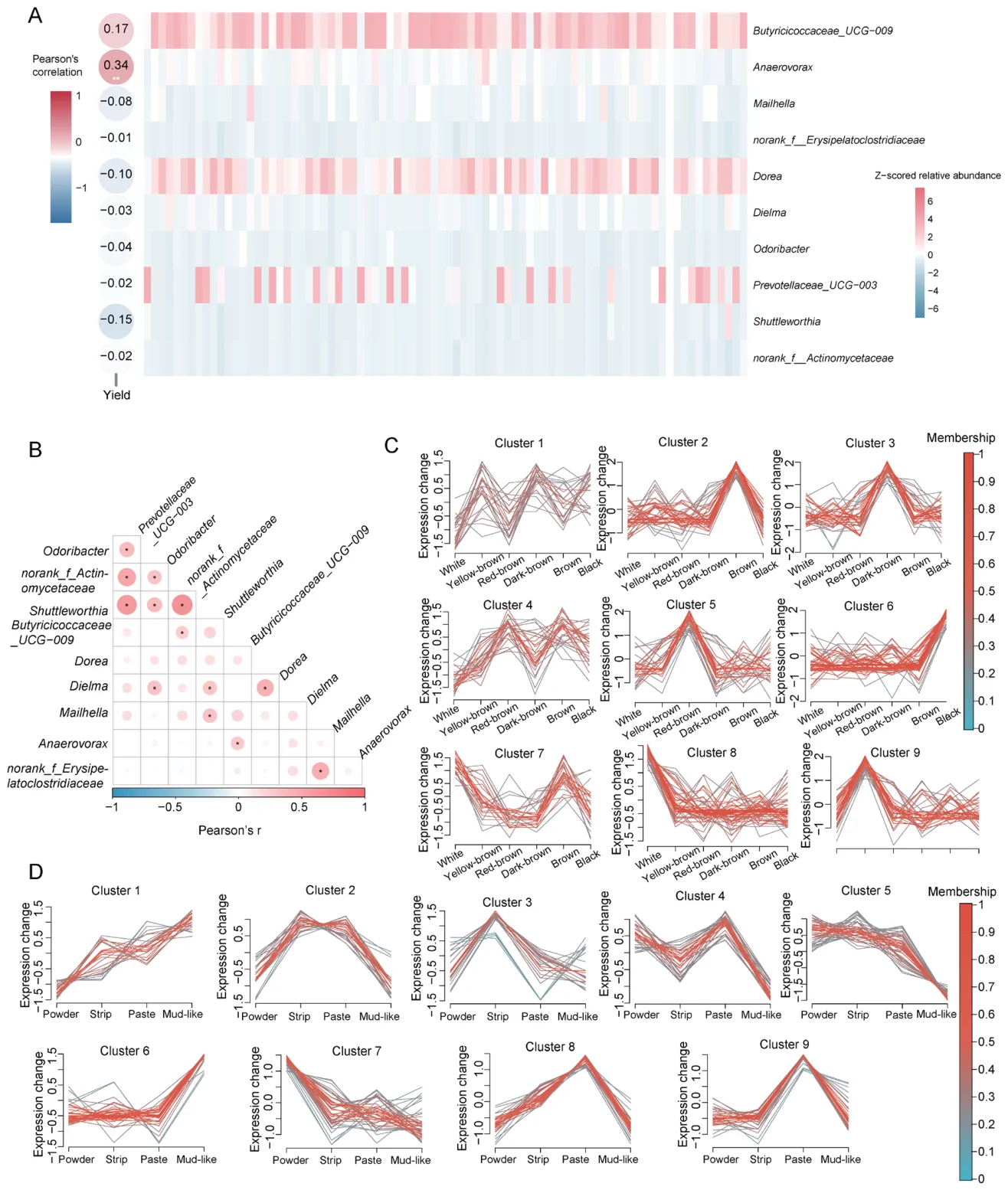
Gut bacteria correlates of musk production in forest musk deer (FMD). (**A**) Pearson correlation between gut bacteria and musk yield (*n* = 83), only taxa that were significantly associated with musk yield at the raw *p* < 0.01 level are displayed. ** indicates significance at BH-FDR-adjusted *p* < 0.05. (**B**) Co-occurrence network of production-associated genera, * means *p* < 0.05. (**C**,**D**) Mfuzz clustering of gut bacteria by expression patterns in color (**C**) and morphology (**D**) groups. Red lines denote high-membership taxa; the Y-axis shows normalized expression values, highlighting trait-specific microbial activity.

The Pearson correlation analysis between gut microbiota and musk production was performed to elucidate gut bacteria influencing musk yield. The six individuals that did not produce musk were excluded; the remaining 83 deer were included in the correlation analysis (Supplementary Table S1). Among the 457 taxa, 10 genus-trait pairs were significantly correlated with the raw value of *p* < 0.05. After BH-FDR multiple-testing correction, only the positive correlation between *Anaerovorax* relative abundance and musk yield remained statistically significant (r = 0.336, adjusted *p* = 0.020); all other genus-yield associations were non-significant after correction (Supplementary Table S6). The relative abundances of *Butyricicoccaceae UCG-009*, *Dorea*, and *Prevotellaceae_UCG-003* were relatively high (Figure 5A). Co-occurrence analysis revealed positive intra-genera correlations: *Prevotellaceae_UCG-003*, *Odoribacter*, *norank_f_Actinomycetaceae*, and *Shuttleworthia* were mutually positively correlated; *norank_f_Actinomycetaceae* correlated with *Butyricicoccaceae UCG-009*; *Mailhella* correlated with *norank_f_Erysipelatoclostridiaceae*; and *Dielma* correlated with *Odoribacter*, *Shuttleworthia*, and *Dorea* (Figure 5B). These patterns suggest synergistic microbial networks modulating musk production.

Gut bacteria exhibited distinct expression patterns linked to musk quality (color, morphology). In the color group: Cluster 8 (e.g., *Prevotellaceae_UCG-001*, *Intestinibacter*, *Actinobacillus*) was highly expressed in white musk. Cluster 9 (e.g., *Succinivibrio*, *Pseudobutyrivibrio*, *Catenibacterium*) was enriched in yellow-brown musk. Cluster 5 (e.g., *Catenisphaera*, *Enterococcus*, *Bifidobacterium*) dominated red-brown musk. Cluster 3 (e.g., *Lachnospiraceae_UCG-008*, *unclassified_f_Bifidobacteriaceae*, *Subdoligranulum*) was active in dark-brown musk. Cluster 2 (e.g., *cc*) characterized brown musk. Cluster 6 (e.g., *Glutamicibacter*, *Microbacterium*, *unclassified_f_Bacillaceae*, *Lachnospiraceae_UCG-002*) was prominent in black musk (Figure 5C). In the morphology group (Figure 5D), Cluster 7 (e.g., *norank_f_Actinomycetaceae*, *Victivallis*, *Lachnospiraceae_FCS020_group*) was highly expressed in powder musk. Cluster 3 (e.g., *Erysipelotrichaceae_UCG-008*, *unclassified_f_Acidaminococcaceae*, *Lachnospiraceae_UCG-002*) dominated strip-shaped musk. Cluster 8 (e.g., *unclassified_p_Bacteroidota*, *norank_f_Eggerthellaceae*, *unclassified_o_Lactobacillales*) and Cluster 9 (e.g., *Bifidobacterium*, *Subdoligranulum*, *unclassified_f_Bifidobacteriaceae*, *Lachnospiraceae_UCG-008*) were enriched in paste-like musk. Cluster 6 (e.g., *unclassified_f_Planococcaceae*, *Streptomyces*, *Glutamicibacter*) characterized mud-like musk.

## 4. Discussion

The gut microbiota is essential for host metabolism, immunity, and intestinal health [41], and its disruption impairs host physiology [42]. However, the link between gut bacteria and musk traits in FMD remains largely unknown. Previous studies have shown that age does not significantly affect gut microbial diversity in adult forest musk deer [43]. Accordingly, we used 89 healthy male FMD (aged 4–12 years) grouped by musk color and morphology, all of which were within the adult plateau phase of musk production, to investigate how gut bacteria influence musk production and quality. This approach addresses critical knowledge gaps in microbiota-mediated mechanisms of musk quality.

### 4.1. Gut Microbiota Composition Is Associated with Musk Color and Moisture Content

Musk quality, defined by color and moisture content, was associated with distinct gut microbial composition patterns. At the phylum level, Firmicutes (71.52%) and Bacteroidota (22.26%) dominated the gut microbiota across all color and morphology groups, consistent with previous reports in FMD [10,30]. The Firmicutes/Bacteroidetes (F/B) ratio ranged from 2.38 in white musk to 4.06 in dark-brown musk and is a well-documented indicator of energy harvesting efficiency in ruminants and related species. In FMD, Firmicutes and Bacteroidota overwhelmingly dominate the gut microbiota, collectively accounting for over 90% of the microbial community [44,45].

Three Firmicutes taxa (*Oscillospiraceae UCG-005*, *unclassified_f_Lachnospiraceae*, *Christensenellaceae_R-7_group*) were dominant at the genus level. *Oscillospiraceae UCG-005* plays regulatory roles in cofactor, vitamin, and energy metabolism [46], has been associated with host immune activity and overall health in the Saiga antelope, and is involved in SCFA synthesis, which benefits host energy metabolism and health [47]. Lachnospiraceae are a core family of anaerobic gut bacteria that ferment complex carbohydrates to produce SCFAs (butyrate and acetate), and play a major role in host health, physiology, and production traits in ruminants [48]. In musk deer, *Lachnospiraceae* abundance increases with age and is associated with optimal dietary energy (11.86 MJ per kg) and protein (13.37%) levels, which promote growth, nutrient absorption, and gut health. Similarly, a dietary crude protein level of 13.37% increases the proportion of *uncultured_bacterium_f_Lachnospiraceae* and improves average daily gain and feed efficiency. Christensenellaceae are key regulators of host metabolism and energy balance through SCFAs production (acetate and butyrate), and are probiotic candidates that improve gut barrier integrity, suppress inflammation, and alleviate metabolic diseases [49,50,51,52]. Increased abundance of *Christensenellaceae R-7 group* improved carbohydrate and lipid metabolism in the rumen of caragana-fed sheep [53], and enhanced adhesion and mucin-utilization capabilities that facilitate gut colonization in pigs [54]. These findings suggest that *Christensenellaceae R-7 group* probably serves as an important candidate taxon for feed efficiency and lipid metabolism. As beneficial gut bacteria, the *Bacteroides* species degrade complex carbohydrates into SCFAs, which benefit colonic health and have potential in treating diseases such as inflammatory bowel diseases and neurological disorders [55,56,57].

In our study, higher F/B ratios were associated with darker-colored and lower-moisture musk, which is considered higher quality [27]. This may reflect the fact that Firmicutes-dominated communities are more efficient at extracting energy from dietary fiber and producing SCFAs, which provide the energetic foundation for steroid and lipid biosynthesis required for musk formation [14,15,58]. However, previous studies in steers have shown that higher F/B ratios are associated with lower feed efficiency [59], suggesting that the relationship between F/B ratio and host productivity may be context-dependent. Furthermore, not all Firmicutes members of Firmicutes share the same functional roles; the specific Firmicutes taxa enriched in high-quality musk groups are known SCFA producers that may be more metabolically beneficial than other Firmicutes members associated with reduced feed efficiency. Future studies integrating metabolomic and transcriptomic data are needed to clarify the functional roles of the F/B ratio in the context of musk production.

No significant difference in α diversity was observed between groups, but β diversity differed significantly, suggesting that microbial community composition is more important than richness. The GMHI showed a significant difference (*p* = 0.03) between the musk-secreting group and non-musk-secreting groups, along with a negative correlation with ACE, indicating potential gut microbiota dysbiosis in the non-musk-secreting group. Therefore, the gut microbiota health index may serve as an indicator of musk secretion potential. Although some studies suggest that captivity stress may lead to non-secretion [27], the exact cause remains unclear.

It should be noted that certain groups in this study had small sample sizes (white musk: *n* = 7; mud-like musk: *n* = 5; non-musk-secreting: *n* = 6), which limits statistical power and increases the risk of false-positive findings. The GMHI should therefore be considered hypothesis-generating rather than confirmatory. While significant differences were observed between some groups, these results should be interpreted with caution and require validation in larger, independent cohorts. Future studies with more balanced group sizes are warranted to confirm these associations. Additionally, we acknowledge that unmeasured age-related factors may still influence the observed associations. Moreover, the cross-sectional nature of this study precludes causal inference; the observed associations may reflect microbial influences on host metabolism, host selection of microbial communities, or both. Future studies with age-matched cohorts, longitudinal designs, or intervention trials are warranted to establish causal relationships.

### 4.2. Differential Taxa and Functional Pathways Underpin Musk Quality Variation

Gut microbial composition differed significantly among musk color and morphology groups, suggesting that microbiota shifts influence host metabolism and endocrine status, thereby affecting musk traits [26]. Multigroup comparisons identified color-associated taxa (*Blautia*, *Phascolarctobacterium*, *Oscillospiraceae UCG-002*, *Eubacterium ruminantium group*) and morphology-associated taxa (*norank_f_UCG-010*, *Parabacteroides*, *Gemmobacter*, *Candidatus_Stoquefichus*). Notably, mud-like musk uniquely enriched *Gemmobacter*, *Glutamicibacter*, *Dyadobacter*, *Rhodococcus*, *Ketogulonicigenium*, and *Nocardioides*, implying roles in moisture-dependent maturation.

Color-associated potential biomarker candidate taxa are potentially linked to SCFA production, lipid/steroid metabolism, and anti-inflammation, which regulate intestinal metabolism, support energy homeostasis, and gut health [60,61,62,63]. *Blautia* is a promising candidate genus for feed additives, with important functions in regulating host immunity and intestinal microecology. *B. hominis* LYH1 down-regulates pro-inflammatory cytokines and inhibits *Salmonella Typhimurium* in macrophages [64]. *B. hominis* HA2291 produces SCFAs and exhibits bile salt hydrolase activity, improving lipid profiles and gut health [65]. Additionally, gut symbiont *B. coccoides* stimulates colonic mucus via Ffar2 [62] and metabolizes tryptophan to increase circulating indole-3-acetic acid (I3AA), thereby reducing inflammation [66,67]. Thus, *Blautia* alleviates inflammation [68], and its metabolites possess immunomodulatory properties [69]. It has been identified as a beneficial bacterium in musk deer [70], and increased abundance correlated with inhibited inflammatory responses [71]. However, its direct impact on musk quality requires further investigation.

*Phascolarctobacterium* produces SCFAs from succinate and pyruvate [72,73], and exhibits anti-inflammatory effects [74]. *P. succinatutens* improves host organ development, intestinal barrier function, and steroid hormone metabolism in pigs [66]. It alleviates inflammation via propionate-mediated TLR4 inhibition and reduces fat deposition by increasing arginine through PI3K/Akt/FOXO3a pathway inhibition in pigs [74]. *P. faecium* is a potential second-generation probiotic that mitigates obesity and metabolic disease by restoring innate immunity via PYY signaling [75,76]. Therefore, *Phascolarctobacterium* is a candidate gut bacterium linking lipid/steroid metabolism and anti-inflammatory capacity, relevant to musk formation and its pharmacological effects [77].

Oscillospiraceae are most abundant in the colon and are considered butyrate producers that maintain intestinal barrier integrity and immune homeostasis [78,79]. *Oscillospiraceae UCG-002* produces SCFA through fiber fermentation, improving immunity and mood [80]. The *Eubacterium_ruminantium_group* is an acetogenic bacterium in the rumen that promotes volatile fatty acid (VFA) synthesis and cholesterol metabolism and converts primary to secondary bile acids [81]. *Parabacteroides* species (e.g., *P. johnsonii*, *P. distasonis*) improve metabolic disorders by converting branched-chain amino acids (BCAAs) to branched-chain short-chain fatty acids (BSCFAs) [81], alleviate obesity and insulin resistance via succinate and secondary bile acids, and strengthen gut barrier integrity [82,83,84]. They also exert anti-inflammatory, immunomodulatory, and anti-cancer effects. For example, *P. distasonis* produces I3AA to suppress bladder cancer through the AhR-FAS axis while enhancing epithelial repair and SCFA production [85].

Morphology-associated potential candidate taxa have diverse metabolic functions and may influence host health and microbiota balance [84,86,87,88]. They probably modulate musk quality through metabolic interactions, although direct functional links to secretion or maturation require validation. *Rhodococcus* degrades aromatic hydrocarbons and halogenated compounds; enhances nutrient utilization and detoxification; and maintains intestinal homeostasis in Tibetan pigs [89,90,91]. *Nocardioides* is an aerobic actinobacterium that degrades aromatic pollutants [87] and metabolizes dimethyl sulfide (DMS) and dimethyl sulfoxide (DMSO), implicating sulfur-containing amino acid metabolism (e.g., cysteine, methionine), which are critical precursors for bioactive musk components [92]. *N. dongkuii* and *N. lijunqiniae*, isolated from the feces of colonized ruminants (Tibetan antelope), produce ε-poly-L-lysine, an antimicrobial peptide [93,94], suggesting that *Nocardioides* may contribute to antimicrobial peptide formation in musk.

*Bacillus* is a key candidate involved in lipid metabolism. *Ketogulonicigenium vulgare* requires co-culture with *Bacillus megaterium* due to metabolic deficiencies during industrial applications [95]. *K. vulgare* possesses 48 genes for extracellular amino acid transporters and 103 for exogenous peptide transport, indicating a robust capacity for scavenging extracellular amino acids [96]. Given that musk polypeptides are known anti-inflammatory components [97] and cell membrane-associated peptides play key roles in musk biosynthesis [98], this strong peptide/amino acid uptake ability suggests that fecal *K. vulgare* and *Bacillus* may be closely associated with the formation of bioactive polypeptides and steroids.

Gut microbial SCFAs (acetate, propionate, butyrate) supply over 70% of host energy and support gut barrier integrity [99], while keystone taxa producing propionate and butyrate boost host production in ruminants [100,101]. ABC transporters enhance nutrient uptake and fermentation efficiency, and transmembrane transport of lipids, sterols, and vitamins, thereby supporting energy harvest, host growth, and musk biosynthesis [11,17,30,102]. In musk deer, SCFAs interact with steroid metabolism to regulate musk secretion, and enhanced energy metabolism creates a favorable environment for aromatic precursor biosynthesis required for high-quality musk pigmentation and maturation [8,15]. Notably, the butyrate-producing commensal UCG-010 is associated with improved lipid metabolism, hormone regulation, and antioxidation in FMD [103]. Additionally, enrichment of amino acid biosynthesis pathways is also important in FMD; active dry yeast supplementation enriches Firmicutes/Bacteroidota and elevates L-cysteine (a precursor for bioactive musk compounds), while a high-energy diet enriches SCFA-producing bacteria (*Akkermansia*, *Lachnospira*) and improves growth [8,11]. Furthermore, gut microbiota modulates steroid hormone levels (testosterone, estradiol, cortisol) via SCFAs, regulating the endocrine environment for normal musk secretion [15]. Collectively, strong lipid- and amino acid- metabolizing capabilities underpin host energy and nitrogen balance, determining resources for high-quality musk production [11,15]. Thus, dark-brown musk and powder groups exhibit microbial signatures primed for high-quality musk, whereas mud-like musk reflects metabolic inefficiency and impaired secretory function. This highlights that a microbiota geared toward efficient nutrient utilization is a prerequisite for optimal musk biosynthesis [8].

### 4.3. Commensal Bacteria Suggest Microbial Synergy in Musk Traits

Beyond exploring gut bacteria-musk quality associations, this study further analyzed correlations between gut microbiota and musk production to identify key bacterial taxa linked to musk yield. The taxa *norank_f_Actinomycetaceae*, *Mailhella*, *Anaerovorax*, and *Prevotellaceae_UCG-003* exhibit synergistic co-occurrence (mutually positive correlations) and their relative abundances correlated positively with musk production. *norank_f_Actinomycetaceae* belongs to Actinobacteria, which are dominant bacteria in musk. Actinobacteria were significantly enriched in mature, high-quality musk and positively associated with better musk quality [16,32]. They play a key metabolic role in antibiotic and terpenoid metabolism, probably contributing to the synthesis of muscone, androstenedione, and antimicrobial substances [10]. *Mailhella* produces organic sulfur-containing metabolites that are important volatile constituents contributing to the characteristic aroma profiles of certain animal secretions and glandular products [104]. This may be highly relevant for understanding its role in the production of malodorous gases in musk. *Anaerovorax* is a strictly anaerobic bacterium that ferments putrescine and other amines to produce SCFAs (such as acetate and butyrate) [105] and contributes to nitrogen metabolism and gut barrier function [106]. In musk deer, the SCFA metabolites may serve as a crucial energy source and help the host improve dietary nitrogen utilization and protein synthesis during musk secretion. *Prevotellaceae_UCG-003* is a predominant ruminant genus that degrades fibers to produce SCFAs, supporting host nutrient absorption and energy harvest [107]. Its enrichment is consistently correlated with improved growth performance, rumen papilla development, and higher production traits, including increased antler yield in red deer, as well as greater body weight and milk yield in other ruminants; it also promotes a healthier gut ecological state [108,109,110,111]. Therefore, these gut bacteria might influence musk traits through their metabolites, such as SCFAs, sulfur-containing volatiles, and terpenoid-related compounds, thereby supporting host energy metabolism, nitrogen utilization, and the biosynthesis of key musk odorants and bioactive components.

Importantly, our findings are based on correlational analyses and PICRUSt2-based functional predictions. Direct measurements of SCFAs, steroid hormones, muscone, and sulfur metabolites were not performed in this study. Thus, the proposed mechanistic links between specific taxa and musk component biosynthesis remain hypothetical and require validation through targeted metabolomics and intervention studies (e.g., probiotic supplementation, dietary modulation, or fecal microbiota transplantation). Additionally, this study was conducted on clinically healthy captive deer under standardized conditions; the extent to which these findings apply to wild populations or diseased individuals remains unknown.

## 5. Conclusions

This study provides the first comprehensive evidence that fecal microbiota composition is associated with both musk quality (color and moisture) and yield in captive male FMD. We observed an elevated F/B ratio in darker-colored and lower-moisture musk by integrating 16S rRNA sequencing with detailed phenotypic characterization of musk traits. Combined with significant β-diversity differences among trait groups, underscores that microbial community composition, rather than alpha diversity alone, is more closely associated with musk quality. We identified that distinct bacterial consortia might be linked to specific musk phenotypes. Aromatic- and sulfur-metabolizing taxa, including *norank_f_Actinomycetaceae*, *Nocardioides*, *Rhodococcus*, and *Mailhella*, were enriched in higher-quality musk and may participate in the biosynthesis of key bioactive components such as muscone, androstenedione, and volatile sulfur compounds. Concurrently, SCFA-producing taxa (such as *Blautia*, *Phascolarctobacterium*, *Oscillospiraceae UCG-005*, *unclassified_f_Lachnospiraceae*, *Anaerovorax*, *Parabacteroides*, *Prevotellaceae_UCG-003*, *Christensenellaceae R-7 group*, and *Bacteroides*) were associated with enhanced fiber degradation and energy supply via SCFA production, potentially supporting steroid synthesis and lipid metabolism required for musk formation. Notably, *Ketogulonicigenium vulgare* was linked to anti-inflammatory polypeptide and steroid pathways, further suggesting a role in the maturation of pharmacologically active musk components.

These findings have practical implications for captive breeding and conservation of FMD. The identified candidate taxa may provide preliminary correlational clues that could potentially inform future research aiming to develop strategies for identifying high-yielding individuals; however, these are not validated biomarkers and require independent experimental confirmation. It is important to emphasize, however, that the observed associations and the predicted functional inferences are not direct metabolite measurements, and these potential applications require validation through interventional studies.

## Data Availability

The data presented in this study are openly available in NCBI SRA with the accession number PRJNA1203306.

## Supplementary Materials

The following supporting information can be downloaded at: https://www.mdpi.com/article/10.3390/vetsci13090957/s1, Supplementary Table S1: Basic information of forest musk deer and musk; Supplementary Table S2: Data summary of forest musk deer feces for 16S rRNA sequencing; Supplementary Table S3: Taxonomic assignment and its ASV abundance in gut microbiome of forest musk deer; Supplementary Table S4: The relative abundance of the top 3 phyla and 4 genus-level bacteria across musk color and morphology groups; Supplementary Table S5: The *p* values of multiple comparison results of the top 2 phyla and 4 genus-level bacterial abundance across musk color and morphology groups. Supplementary Table S6: Pearson correlation analysis between gut bacterial genera and musk yield in forest musk deer.

## Abbreviations

FMD	Forest musk deer
F/B ratio	Firmicutes to Bacteroidetes ratio
SCFA	Short-chain fatty acid
16S rRNA	16S ribosomal ribonucleic acid
ASV	Amplicon sequence variant
ACE	Abundance-based coverage estimator
PCoA	Principal coordinate analysis
GMHI	Gut Microbiome Health Index
ANOVA	Analysis of variance
KEGG	Kyoto Encyclopedia of Genes and Genomes
PCR	Polymerase chain reaction
DNA	Deoxyribonucleic acid
PICRUSt2	Phylogenetic Investigation of Communities by Reconstruction of Unobserved States 2
FDR	False discovery rate
PERMANOVA	Permutational multivariate analysis of variance
OUT	Operational taxonomic unit
